# Infrared Spectroscopy in Differential Diagnosis of Pulmonary Embolism

**DOI:** 10.17691/stm2020.12.3.08

**Published:** 2020-06-28

**Authors:** O.V. Krasnikova, S.V. Nemirova, A.P. Medvedev, A.S. Gordetsov

**Affiliations:** Associate Professor, Department of General Chemestry; Privolzhsky Research Medical University, 10/1 Minin and Pozharsky Square, Nizhny Novgorod, 603005, Russia; Associate Professor, Department of Hospital Surgery named after B.A. Korolyov; Privolzhsky Research Medical University, 10/1 Minin and Pozharsky Square, Nizhny Novgorod, 603005, Russia; Professor, Department of Hospital Surgery named after B.A. Korolyov; Privolzhsky Research Medical University, 10/1 Minin and Pozharsky Square, Nizhny Novgorod, 603005, Russia; Professor, Head of the Department of General Chemestry Privolzhsky Research Medical University, 10/1 Minin and Pozharsky Square, Nizhny Novgorod, 603005, Russia

**Keywords:** infrared spectroscopy, pulmonary embolism, PE, differential diagnosis of pulmonary diseases.

## Abstract

**The aim of the study** was to assess the effectiveness of infrared spectroscopy for verification of pulmonary embolism (PE) and a number of similar diseases.

**Materials and Methods.:**

Infrared spectroscopy was used to investigate blood serum of 19 healthy volunteers and 30 patients with intraoperatively confirmed PE as well as with chronic obstructive pulmonary disease (COPD) (n=10), pneumonia (n=10), tuberculosis (n=10), lung abscess (n=10) and lung cancer (n=10), acute disorder of cerebral circulation (ADCC) (n=10), ischemic heart disease (IHD) (n=10). Peak height ratios of absorption band were taken as diagnostic parameters (cm^–1^/сm^–1^): P_1_ — 1160/1165; P_2_ — 1165/1070; P_3_ — 1165/1150; P_4_ — 1165/1050; P_5_ — 1100/1050; P_6_ — 1025/1165. These parameters of IR spectrum are significant for the given nosology.

**Results.:**

The calculated indicators have demonstrated statistically significant difference of IR spectra parameters for the studied nosologies (p<0.001) even on the small samples supplementing each other and enabling step-by-step exclusion of lung abscess and pulmonary tuberculosis, COPD and pneumonia, cancer, IHD, ADCC, and PE.

The presented radar charts, built with consideration of the values of all peak height ratios of the absorption bands with diagnostically significant maxima, provided the possibility to visualize the IR profiles making the differentiation of PE and its clinical analogs not only more objective and reliable but also more explicit and compelling.

**Conclusion.:**

Infrared spectroscopy is a potentially effective method of PE differential diagnosis. Sample expansion will allow researchers to evaluate the sensitivity and specificity of this technique compared to the existing standard schemes of PE verification.

## Introduction

A wide prevalence of pulmonary embolism (PE) associated with a high rate of risk factors for venous thrombosis, multiple complications, and significant probability of unfavorable disease outcome dictate the necessity to improve methods of its verification. The existing Russian and world standards prescribe to define the probability of PE in case of acute pain in the chest, loss of consciousness, breathlessness, and hemoptysis, however, symptoms often found in PE may also be similar to those manifested in dissecting aneurysm of the thoracic aorta, ischemic heart disease (IHD), pneumothorax, tuberculosis, obstructive, oncologic, purulent destructive diseases of the lungs, acute disorder of cerebral circulation (ADCC), and also in intercostal neuralgia with a highly prominent pain syndrome [1–4].

When attempting to evaluate the PE probability clinically, diagnostic errors occur rather often resulting in late rendering of specialized medical assistance, patient death, or formation of progressing complications also leading to fatal outcome [5–8]. In spite of the fact that chronic post-embolic pulmonary hypertension morbidity is 0.1–9.1% during the first two years after the episode of symptomatic PE [9], it is fixed in the International Registry that 74.8% of patients with persistent increased pressure in the pulmonary trunk had acute PE in the past history [10], and data are available on a wider prevalence of this syndrome: occurring in 5 people per1 million population annually [11].

The development of chronic post-embolic pulmonary hypertension has been proved [12–14] to worsen not only patients quality of life but the disease prognosis as well, to require lifelong systemic therapy or operative intervention under extracorporeal circulation and hypothermia. Lethality and complications place a supplementary economic burden on the public health service and the state in general which, in particular, was clearly demonstrated in the recent investigations carried out in Russia and confirmed by the foreign authors [15–17].

The situation is still more aggravated by a frequent combination of the mentioned diseases since oncopathology and injury of bronchopulmonary and cardiovascular systems lead to hypercoagulation and blood flow deceleration and, in case of acute inflammatory reaction and a destructive process, to the damage of the vascular wall, i.e. to the formation of all three components of Virchow’s triad: an integral component of thrombus formation [18–20].

Methods and criteria for differential monopathology diagnosis have been proposed and are being developed, among which laboratory techniques are evolving most actively [21]. Spectral investigations are considered rather perspective in this respect. Infrared (IR) spectroscopy, in particular, is part of molecular optical spectroscopy studying absorption spectra and reflections of electromagnetic radiation in IR region, i.e. in the range of wavelengths from 10^–6^ to 10^–3^ m. This objective method does not practically require expensive reagents and consumables for its application and allows specialists to identify presence and dynamics of metabolite concentration showing the appropriate pathology of the specimen biochemical composition.

**The aim of the investigation** was to study the potentials of IR spectroscopy to verify pulmonary embolism and a number of similar clinical diseases.

## Materials and Methods

The study was carried out jointly at the clinics of the Department of Hospital Surgery named after B.A. Korolyov and the Department of General Chemistry of Privolzhsky Research Medical University (Nizhny Novgorod) in two stages:

in *the first stage*, IR spectra of blood serum from 19 healthy volunteers and 30 patients with intraoperatively confirmed PE were obtained and compared;

in *the second stage*, IR spectra of blood serum were obtained and compared in PE and chronic obstructive pulmonary disease (COPD) (n=10), pneumonia (n=10), tuberculosis (n=10), lung abscess (n=10) and lung cancer (n=10), ADCC (n=10), IHD (n=10).

The work was done in compliance with the Declaration of Helsinki (2013) and approved by the Ethics Committee of Privolzhsky Research Medical University. Informed written consent was obtained from each patient.

5 ml of blood for the complex of investigations was collected from the cubital vein of each patient in the morning on their admission and centrifuged for 15 min at 1000 rpm. The separated serum (1.0 ml) was dried in the dry heat oven in the Petri dish at 25°С for 24 h. The solid serum residual was minced and suspended in liquid paraffin. The IR spectra of the dry blood serum were obtained using spectrophotometers SPECORD 75 IR and М80 (Carl Zeiss, Germany) with a photometric error of 0.2%.

At first, a height of absorption band peaks with maxima at 1165, 1160, 1150, 1100, 1070, 1050, 1025 cm^–1^ was determined. Then values of peak height ratios of the absorption bands (сm^–1^/сm^–1^) were calculated: P_1_ — 1160/1165; P_2_ — 1165/1070; P_3_ — 1165/1150; P_4_ — 1165/1050; P_5_ — 1100/1050; P_6_ — 1025/1165.

## Results and Discussion

**Figure 1:**
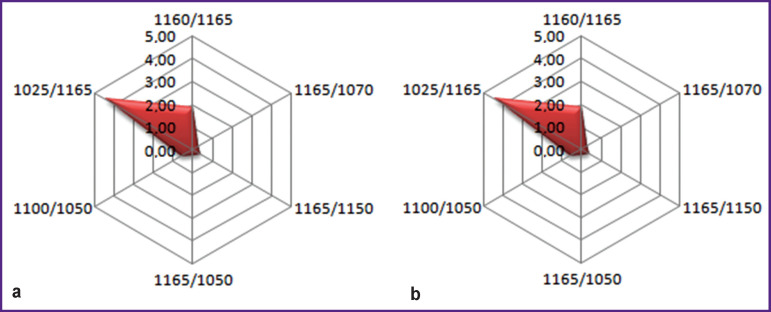
Diagnostic profile obtained by analyzing the results of IR spectroscopy of the blood serum in norm (а) and in pulmonary embolism (b)

The mathematically processed results of the IR spectra of the blood serum taken from the healthy volunteers and PE patients were presented in the form of a radar chart (Figure 1).

The obtained diagnostically significant IR spectra of the blood serum enabled us to develop the method of PE diagnosis based on the differentiation of serum molecular spectra in the range of 1200–1000 cm^–1^ wave numbers (in the IR range) [22].

Next, the IR blood serum spectra of the patients with COPD, pneumonia, tuberculosis, lung abscess, lung cancer, ADCC, IHD were obtained and transformed mathematically into the radar chart according to the same parameters. Thereafter, a comparative analysis of the radar charts of all mentioned nosologies with differentially diagnostic profile of PE was performed.

**Figure 2:**
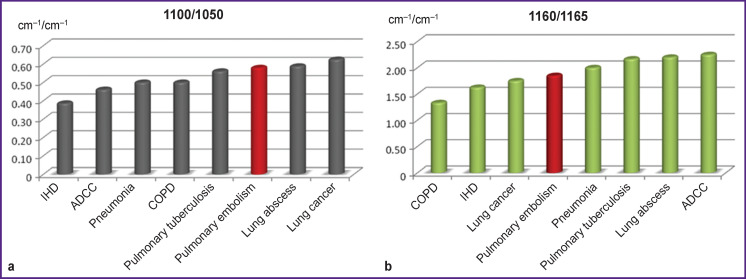
Comparison of 1100/1050 (а) and 1160/1165 (b) ratios of IR spectrum absorption bands in patients with pulmonary embolism and clinically similar diseases

The comparison of 1100/1050 and 1160/1165 ratios demonstrates the differences of values across all examined diseases (p<0.001) and confirms indirectly the existence of common processes in the pathogenesis of all these diseases (Figure 2).

**Figure 3:**
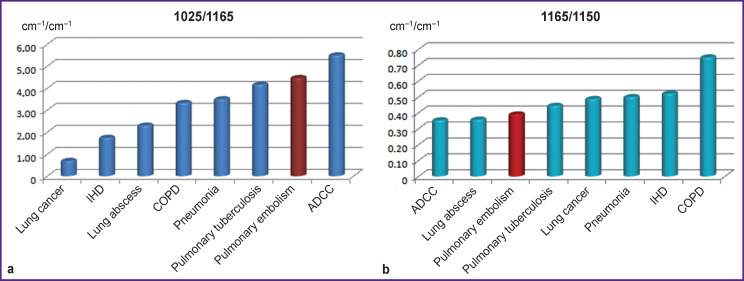
Comparison of 1025/1165 (а) and 1165/1150 (b) ratios of IR spectrum absorption bands in patients with pulmonary embolism and clinically similar diseases

The difference of 1025/1165 ratio (Figure 3 (а)) was the most prominent for IR serum spectra of IHD, COPD, PE, and ADCC (p<0.001) and enabled the differentiation of lung abscess and pulmonary tuberculosis (p<0.001) but was less informative for COPD and pneumonia which would be preferably defined by 1165/1150 ratio, more typical in chronic bronchial obstruction (p<0.001) (Figure 3 (b)).

**Figure 4:**
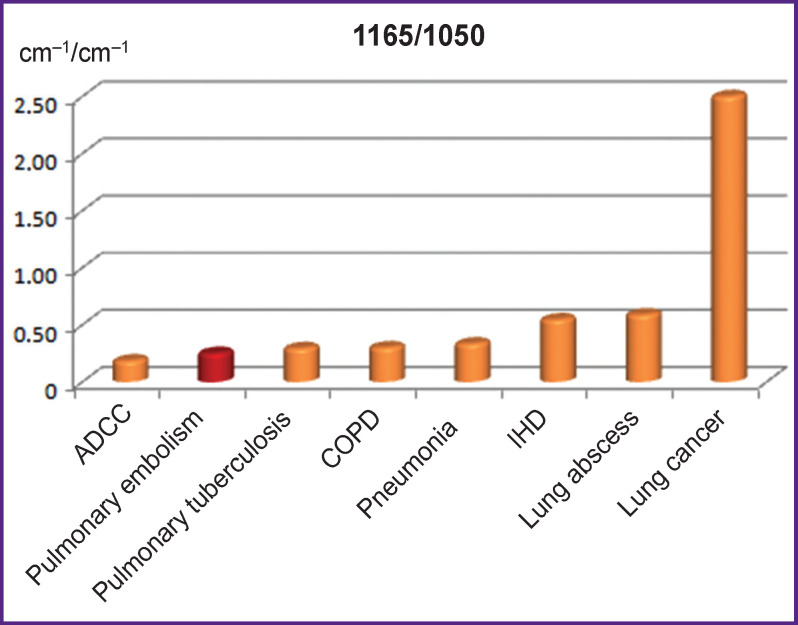
Comparison of 1165/1050 ratios of IR spectrum absorption bands in patients with pulmonary embolism and clinically similar diseases

A high value of 1165/1050 ratio allowed us actually to suspect oncopathology (p<0.001) (Figure 4).

**Figure 5:**
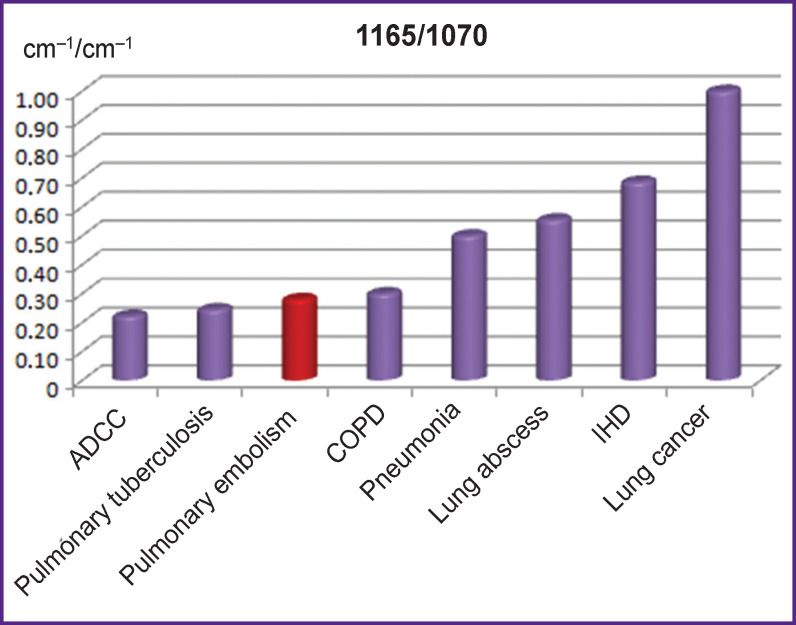
Comparison of 1165/1070 ratios of IR spectrum absorption bands in patients with pulmonary embolism and clinically similar diseases

The 1165/1070 ratio showed also a maximal value in lung cancer (p<0.001) but unlike the previous charts demonstrated clearly the difference between COPD and pneumonia (p<0.001) and allowed us to differentiate PE from IHD (p<0.001) (Figure 5).

**Figure 6:**
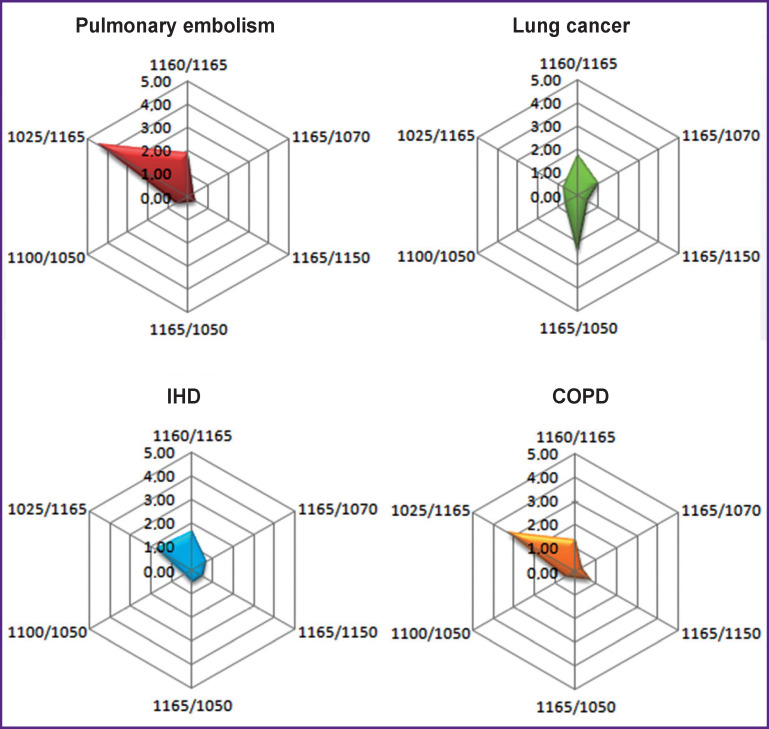
Differentially diagnostic profiles of IR spectra for pulmonary embolism, IHD, COPD, and lung cancer

The chart built with consideration of all parameters became an illustrative differential diagnostic criterion for the diseases during verification of which the diagnostic errors are often encountered (Figure 6).

The current standards of PE diagnosis prescribe to define a D-dimer level, the normal values of which are considered a significant diagnostic criterion for the low risk of thromboembolism, however, its level rises in DIC syndrome, sepsis, and local inflammatory process, for example, in pneumonia, oncopathology, recent operative intervention, or in case of extensive hematoma, hepatic cirrhosis, and myocardial infarction [23, 24]. The diagnostically significant levels of the brain or pro-brain natriuretic peptides as well as troponin I or T are the markers of heart failure in the first place and will also rise in IHD, severe COPD, or bilateral pneumonia with the development of right ventricular insufficiency [25, 26], i.e. are considered to be criteria for the state severity but do not allow for the verification of its etiology [27].

In this work, the unique IR spectral characteristics (positions of the band maxima, their half-width, intensity) of molecules generated in the course of the pathologic metabolism initiated by PE constitute the basis of the differential diagnosis of PE and clinically similar diseases, therefore, the obtained values are distinguished by their high level individuality that makes them valuable for identifying the presence of organic compound combinations.

Despite a small sample volume for each illness and great heterogeneity of parameters within each group such as intensity of the main clinical symptoms and findings obtained by the laboratory and instrumental investigation methods, the calculated indicators have demonstrated a statistically significant difference between the IR spectra for the examined nosologies supplementing each other and enabling step-by-step exclusion of lung abscess and pulmonary tuberculosis, COPD and pneumonia, cancer, IHD, ADCC, and PE.

The presented radar charts built with consideration of the values of all peak height ratios of the absorption bands with diagnostically significant maxima provided the possibility to visualize the IR profiles having made the differentiation of PE and its clinical analogs not only more objective and reliable but also more explicit and compelling.

## Conclusion

In the course of the pilot work, reliable results have been obtained demonstrating potential effectiveness of IR spectroscopy and, consequently, the appropriateness of its application in the differential diagnosis of pulmonary embolism. More extensive samples will allow researchers to evaluate the sensitivity and specificity of this technique compared to the existing standard schemes of pulmonary embolism verification.
